# Alzheimer diseases CSF biomarkers correlate with systemic biomarkers in a cohort of Hispanics

**DOI:** 10.1002/alz.084930

**Published:** 2025-01-09

**Authors:** Alcibiades E Villarreal, Diana C Oviedo, Maria B Carreira, Giselle Rangel, Pahola Araujo, Ricardo Williams de Roux, Rainier Rodríguez Balaguer, Marilisa Díaz, Nelson Novarro‐Escudero, David Alberto Anguizola Tamayo, Julio Villarreal, David Dondis, Aron Benzadon, Gabrielle B Britton

**Affiliations:** ^1^ Sistema Nacional de Investigación (SNI), Panamá, Panamá Panama; ^2^ Instituto de Investigaciones Científicas y Servicios de Alta Tecnología (INDICASAT AIP), Centro de Neurociencias, Panamá, Panamá Panama; ^3^ Universidad Santa María La Antigua, Panamá, Panamá Panama; ^4^ Hospital Pacífica Salud, Panamá, Panamá Panama; ^5^ Hospital Paitilla, Panamá, Panamá Panama; ^6^ Servicio de Neurología, CIDELAS‐CSS, Panamá, Panamá Panama

## Abstract

**Background:**

We are conducting a biomarker‐based study to describe the demographic and biomarker profiles of Panamanians. The objective of this report is to present the main findings on CSF, genetic and serological biomarkers’ distribution and their association with cognition in a cohort of elderly people in Panama.

**Method:**

Informed consent was applied to all participants, demographic data, medical history, and neuropsychological and functional status were collected, and non‐fasting blood samples were obtained. We performed a cross‐sectional analysis of aged individuals for blood and CSF biomarkers and Apolipoprotein E (ApoE) expression (N=45). The following measures were obtained: MMSE, Global Deterioration Scale, and Geriatric Depression Scale 30‐item version. The following biomarkers in CSF were analyzed: Ab1–42, T‐tau and P‐tau181.

**Result:**

We performed group comparisons and analyzed in‐group correlations between blood‐based and CSF biomarkers. Group differences across three CFS profile categories (normal, plausible AD, and AD) were calculated using ANOVA. The Innotest Amyloid Tau Index (IATI) was used to assign 45 biochemical CSF profiles into: normal CSF profile (n=19), plausible AD profile (n=18) and AD profile (n=8). Groups did not differ with respect to age, sex, or education level. AD group expressed at least one copy of the allele 4 of ApoE significantly more than normal and plausible AD profile groups (p=0.017 and 0.035, respectively). Significant correlation between IL‐18 and Ab1–42 was observed in normal group, and no correlations were found between blood and CSF markers in plausible AD group. The greatest number of significant correlations was found in AD group: B2M, CRP, Eotaxin3, PPY, SAA, TARC correlated with P‐tau181; IL‐10, IL‐7 with Ab1–42; and FVII with T‐tau.

**Conclusion:**

In this analysis we observed a strong correlation of systemic biomarkers with CSF profiles (IATI profiles), mainly in the Alzheimer's disease group. Blood‐based biomarkers might facilitate diagnosis and clinical design in the future, but further validation is required.

